# Supplementary material for: Gender-Dependent Associations between Serum Betatrophin Levels and Lipoprotein Subfractions in Diabetic and Nondiabetic Obese Patients
Source: Int J Mol Sci. 2023 Nov 19;24(22):16504. doi: 10.3390/ijms242216504 (PMC10671489; doi:10.3390/ijms242216504)
Supplement: Supplementary file 1 [file ijms-24-16504-s001.zip › ijms-2726929-supplementary.pdf]

Supplementary Table S1. Pearson's correlations of betatrophin with laboratory parameters in enrolled study groups.

	T2D		NDO		Controls	
Variable	r	p	r	p	r	p
Age (yrs)	-0.110	0.447	-0.226	0.060	0.039	0.789
Body mass index (kg/m^2)	**0.346**	**0.017**	0.195	0.114	0.092	0.567
Waist circumference (cm)	**0.366**	**0.024**	**0.262**	**0.049**	0.275	0.105
log10 fasting glucose (mmol/L)	0.116	0.442	0.229	0.079	-0.155	0.289
HbA1C (%)	0.044	0.781	0.042	0.773	0.279	0.070
log10 insulin (mU/L)	**0.797**	**<0.001**	0.233	0.076	0.454	0.078
log10 hsCRP (mg/L)	0.387	0.051	0.093	0.499	0.074	0.687
log10 triglyceride (mmol/L)	**0.400**	**0.039**	0.130	0.341	**0.408**	**0.020**
Total cholesterol (mmol/L)	0.149	0.317	0.227	0.076	0.214	0.140
Apolipoprotein-AI (g/L)	-0.081	0.677	-0.125	0.324	-0.057	0.724
LDL subfraction test						
VLDL (%)	0.199	0.164	0.140	0.247	**0.308**	**0.031**
IDL (%)	**-0.307**	**0.030**	-0.150	0.216	**-0.395**	**0.005**
Large LDL (%)	0.118	0.415	0.100	0.412	**0.367**	**0.010**
Small LDL (%)	0.200	0.164	**0.324**	**0.006**	**0.341**	**0.017**
Mean LDL size (nm)	-0.249	0.081	**-0.317**	**0.008**	**-0.411**	**0.003**
HDL subfraction test						
HDL-1 (%)	-0.102	0.490	**-0.252**	**0.036**	-0.143	0.328
HDL-2 (%)	**-0.308**	**0.033**	**-0.354**	**0.003**	**-0.295**	**0.040**
HDL-3 (%)	**-0.356**	**0.016**	**-0.261**	**0.029**	**-0.300**	**0.036**
HDL-4 (%)	-0.271	0.062	-0.156	0.198	**-0.346**	**0.015**
HDL-5 (%)	-0.164	0.267	-0.046	0.704	0.002	0.987
HDL-6 (%)	0.185	0.208	0.155	0.200	0.211	0.146
HDL-7 (%)	**0.379**	**0.008**	**0.276**	**0.021**	**0.286**	**0.047**
HDL-8 (%)	**0.333**	**0.021**	**0.303**	**0.011**	**0.304**	**0.034**
HDL-9 (%)	0.269	0.064	**0.273**	**0.022**	**0.317**	**0.027**
HDL-10 (%)	0.169	0.252	0.221	0.067	**0.355**	**0.012**
Large HDL (%)	**-0.327**	**0.020**	**-0.339**	**0.004**	**-0.297**	**0.039**
Intermediate HDL (%)	0.058	0.690	0.118	0.330	0.078	0.596
Small HDL (%)	0.240	0.093	**0.288**	**0.016**	**0.351**	**0.013**

Abbreviations: HbA1C, hemoglobin A1C; HDL, high-density lipoprotein; hsCRP, high-sensitivity C-reactive protein; IDL, intermediate-density lipoprotein; LDL, low-density lipoprotein; NDO, nondiabetic obese patients; T2D, patients with type 2 diabetes; VLDL, very low-density lipoprotein.

Notes: Serum betatrophin concentrations were measured by ELISA and lipoprotein subfractions were detected by Lipoprint® acrylamide gel electrophoresis. Nonnormally distributed data were transformed logarithmically before Pearson's correlation analysis. Significant data ($p<0.05$) are bolded.

Supplementary Table S2. Pearson's correlations of conventional cardiovascular risk factors with lipoprotein subfractions in all subjects.

	Age (yrs)		BMI (kg/m^2)		log10 hsCRP (mg/L)		log10 insulin (mU/L)		HOMA-IR	
Variable	r	p	r	p	r	p	r	p	r	p
LDL subfraction test										
VLDL (%)	0.005	0.940	**0.171**	**0.015**	0.126	0.127	0.127	0.162	-0.015	0.887
IDL (%)	-0.091	0.183	-0.065	0.356	**-0.308**	**<0.001**	-0.004	0.966	-0.162	0.126
Large LDL (%)	0.050	0.461	**0.244**	**<0.001**	**0.419**	**<0.001**	0.021	0.822	0.111	0.295
Small LDL (%)	**0.139**	**0.041**	-0.024	0.737	0.121	0.143	0.069	0.448	**0.240**	**0.022**
Mean LDL size (nm)	-0.167	0.014	-0.081	0.251	**-0.221**	**0.007**	-0.138	0.128	**-0.244**	**0.020**
HDL subfraction test										
HDL-1 (%)	-0.002	0.983	**0.278**	**<0.001**	**-0.181**	**0.027**	-0.146	0.110	-0.136	0.198
HDL-2 (%)	0.033	0.635	**-0.352**	**<0.001**	-0.143	0.083	**-0.308**	**<0.001**	**-0.243**	**0.020**
HDL-3 (%)	0.015	0.833	**-0.249**	**<0.001**	-0.135	0.099	**-0.289**	**0.001**	**-0.248**	**0.018**
HDL-4 (%)	-0.089	0.194	**-0.181**	**0.010**	-0.147	0.073	**-0.289**	**0.001**	**-0.288**	**0.006**
HDL-5 (%)	**-0.199**	**0.003**	-0.028	0.696	-0.060	0.470	**-0.204**	**0.025**	**-0.238**	**0.023**
HDL-6 (%)	-0.105	0.125	**0.256**	**<0.001**	0.153	0.063	0.116	0.204	0.150	0.156
HDL-7 (%)	-0.020	0.773	**0.319**	**<0.001**	0.109	0.187	**0.254**	**0.050**	**0.260**	**0.013**
HDL-8 (%)	0.016	0.821	**0.159**	**0.024**	0.017	0.835	**0.139**	**0.011**	0.087	0.412
HDL-9 (%)	0.022	0.750	0.091	0.197	-0.035	0.671	**0.230**	**0.011**	0.168	0.113
HDL-10 (%)	0.112	0.103	**0.279**	**<0.001**	**0.244**	**0.003**	**0.379**	**<0.001**	**0.326**	**0.002**
Large HDL (%)	0.018	0.788	**-0.350**	**<0.001**	**-0.183**	**0.026**	**-0.308**	**<0.001**	**-0.247**	**0.019**
Intermediate HDL (%)	**-0.211**	**0.002**	**0.181**	**0.010**	0.044	0.596	-0.060	0.509	-0.055	0.605
Small HDL (%)	0.089	0.120	**0.255**	**<0.001**	**0.178**	**0.030**	**0.361**	**<0.001**	**0.297**	**0.004**

Abbreviations: BMI, body mass index; HDL, high-density lipoprotein; hsCRP, high-sensitivity C-reactive protein; HOMA-IR, homeostasis model assessment insulin resistance; IDL, intermediate-density lipoprotein; LDL, low-density lipoprotein; VLDL, very low-density lipoprotein.

Notes: Nonnormally distributed data were transformed logarithmically before Pearson's correlation analysis. Significant data (p<0.05) are marked in bold.
